# Critical Role of Zinc in a New Murine Model of Enterotoxigenic Escherichia coli Diarrhea

**DOI:** 10.1128/IAI.00183-18

**Published:** 2018-06-21

**Authors:** D. T. Bolick, P. H. Q. S. Medeiros, S. E. Ledwaba, A. A. M. Lima, J. P. Nataro, E. M. Barry, R. L. Guerrant

**Affiliations:** a Division of Infectious Disease and International Health, University of Virginia School of Medicine, Charlottesville, Virginia, USA; b Institute of Biomedicine, Federal University of Ceara, Fortaleza, CE, Brazil; c Department of Microbiology, University of Venda, Thohoyandou, Limpopo Province, South Africa; d Department of Pediatrics, University of Virginia School of Medicine, Charlottesville, Virginia, USA; e Center for Vaccine Development, Microbiology and Immunology, University of Maryland School of Medicine, Baltimore, Maryland, USA; University of California San Diego School of Medicine

**Keywords:** ETEC, murine model, zinc, LT and ST, enteropathy, diarrhea

## Abstract

Enterotoxigenic Escherichia coli (ETEC) is a major cause of traveler's diarrhea as well as of endemic diarrhea and stunting in children in developing areas. However, a small-mammal model has been badly needed to better understand and assess mechanisms, vaccines, and interventions. We report a murine model of ETEC diarrhea, weight loss, and enteropathy and investigate the role of zinc in the outcomes. ETEC strains producing heat-labile toxins (LT) and heat-stable toxins (ST) that were given to weaned C57BL/6 mice after antibiotic disruption of normal microbiota caused growth impairment, watery diarrhea, heavy stool shedding, and mild to moderate intestinal inflammation, the latter being worse with zinc deficiency. Zinc treatment promoted growth in zinc-deficient infected mice, and subinhibitory levels of zinc reduced expression of ETEC virulence genes *cfa1*, *cexE*, *sta2*, and *degP* but not of *eltA in vitro*. Zinc supplementation increased shedding and the ileal burden of wild-type (WT) ETEC but decreased shedding and the tissue burden of LT knockout (LTKO) ETEC. LTKO ETEC-infected mice had delayed disease onset and also had less inflammation by fecal myeloperoxidase (MPO) assessment. These findings provide a new murine model of ETEC infection that can help elucidate mechanisms of growth, diarrhea, and inflammatory responses as well as potential vaccines and interventions.

## INTRODUCTION

Enterotoxigenic Escherichia coli (ETEC) is one of the most common causes of childhood diarrhea in developing countries and is a leading cause of diarrhea deaths in infants (1). In the GEMS and MALED studies, ETEC was found to be one of the top 4 most common pathogens in children aged 0 to 59 months and was associated with diarrhea and increased risk of death (2, 3). Additionally, Lamberti et al. reported that ETEC is the leading cause of diarrhea-associated mortality and morbidity (followed by Shigella) in children who are >5 years of age in both South Asia and Africa (4).

ETEC infections are also the leading cause of traveler's diarrhea (followed by Campylobacter infections) (5). The incidence of traveler's diarrhea is estimated at 20% to 50% of all travelers to developing countries (6). ETEC infection in adults usually occurs 1 to 3 days after exposure and lasts from 3 to 5 days. Common symptoms are watery diarrhea, abdominal cramping, nausea, vomiting, and fever (7, 8).

ETEC virulence is associated with colonization of the small intestine and production of one or more toxins that induce secretion of chloride, sodium, and water into the lumen (9). The two major classes of toxins, the heat-labile toxins (LT) and heat-stable toxins (ST), act by stimulating expression of adenylate and guanylate cyclase, respectively (10, 11). ETEC strains may have one or both of the LT and ST (12). The toxins are encoded on large plasmids which also encode most of the known ETEC colonization factors (CFs) (13). There is a large variety of colonization factors. Most are fimbrial or fibrillary proteins and consist of a single antigen (colonization factor antigen I [CFA/I]) or of multiple fibrillary “colonization surface antigens” (which currently number 1 to 21, including colonization surface antigen 1 [CS1] to CS3, constituting CFA/II, and CS4 to CS6, constituting CFA/IV) (14). Sensitive quantitative PCR (qPCR) arrays have recently been developed to identify ETEC colonization factors (15). Both the toxins and colonization factors of ETEC have been targeted for vaccine development (16–18).

In the current report, we describe a robust, small-animal model of ETEC infection in young mice exhibiting diarrhea, weight loss, and mild to moderate intestinal inflammation. We also investigate the impact of zinc deficiency as well as zinc treatment on disease outcomes. This model will be valuable in testing potential interventions as well as ETEC vaccine candidates.

## RESULTS

### Enterotoxigenic Escherichia coli infection causes weight loss and diarrhea.

**FIG 1:**
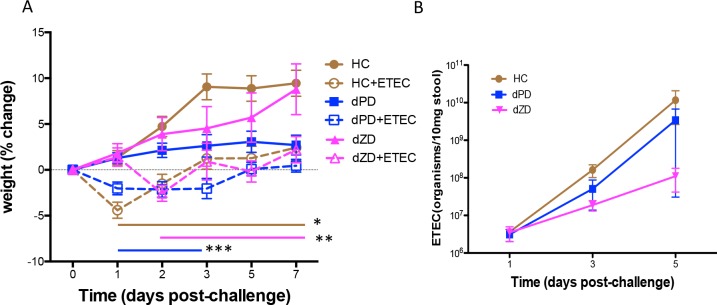
Effects of ETEC strain H10407 infection in antibiotic-pretreated mice fed a nourished diet (HC), a protein-deficient diet (dPD), or a zinc-deficient diet (dZD). Mice were fed one of the three diets (HC, dPD, or dZD) for 2 weeks and pretreated with an antibiotic cocktail prior to infection with 10^8^ CFU of ETEC strain H10407. (A) Body weight change postchallenge. Daily weight measures were collected after infection. *, *P* < 0.01 (HC versus HC plus ETEC during days 1 to 7 postinfection); **, *P* < 0.005 (dZD versus dZD plus ETEC during days 2 to 7 postinfection) (*n* = 8/group); ***, *P* < 0.05 (dPD versus dPD plus ETEC during days 1 to 3 postinfection). (B) Stool ETEC H10407 shedding. Quantitative PCR was performed using DNA samples that had been extracted from stool samples from ETEC-infected mice (*n* = 8/group).

As shown in Fig. 1A, mice infected with ETEC lost weight as early as day 1 postinfection (pi) (*, *P* < 0.01 [standard rodent “house chow” {HC} diet versus HC plus ETEC during days 1 to 7]; **, *P* < 0.005 [defined protein source diet without zinc {dZD} versus dZD plus ETEC during days 2 to 7]), and the mice fed HC or dZD developed watery diarrhea. While mice fed with a defined reduced-protein diet (dPD) did not develop diarrhea, they did have significant weight loss at 3 days postinfection (***, *P* < 0.05 [dPD versus dPD plus ETEC during days 1 to 3]).

### Enterotoxigenic E. coli shedding in stool.

Antibiotic disruption of resident microbiota was required for ETEC colonization in our mouse model as there was no detectable level of ETEC in stool following infection in non-antibiotic-treated mice (data not shown). However, in mice pretreated with the antibiotic cocktail described in Materials and Methods, ETEC was shed (at 7 to 10 logs/10 mg stool) in mouse feces during days 1, 3, and 5 postinfection (Fig. 1B), effects that were not increased under conditions of zinc or protein deficiency.

### Enterotoxigenic E. coli increases levels of intestinal inflammatory biomarkers in mice.

**FIG 2:**
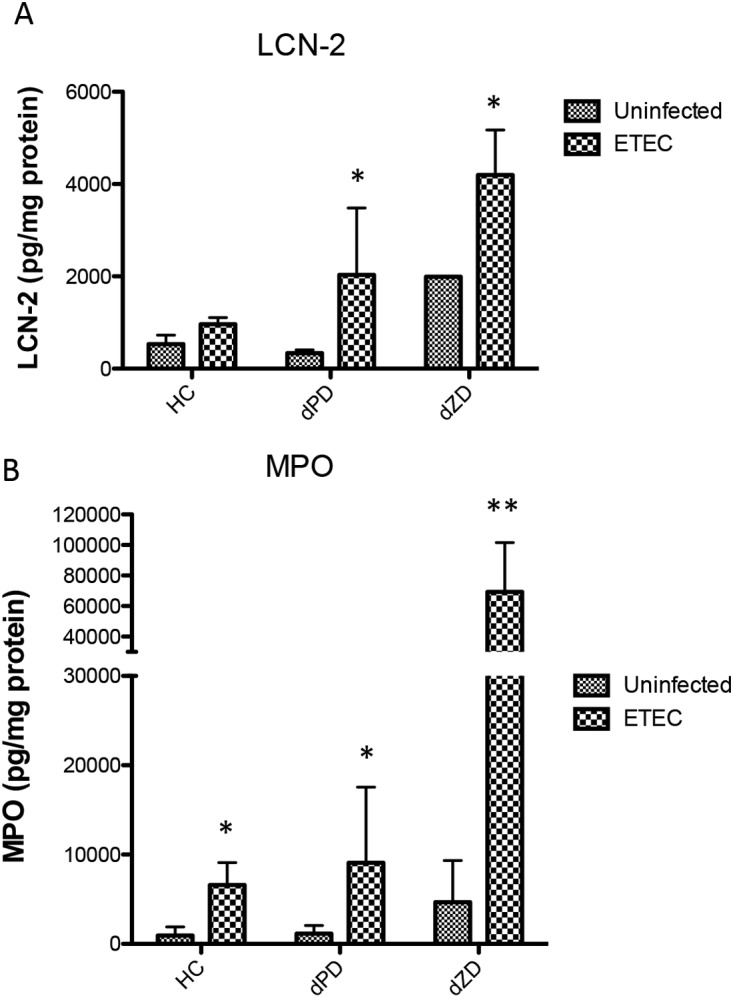
Intestinal inflammation induced by ETEC strain H10407 infection in antibiotic-pretreated mice fed a nourished diet (HC), a protein-deficient diet (dPD), or a zinc-deficient diet (dZD). Protein extracts were obtained from fecal samples at day 2 postinfection by lysing fecal specimens in RIPA buffer. Supernatants from the lysates were used for protein quantification and specific ELISAs for evaluation of myeloperoxidase (MPO) or lipocalin (LCN-2). (A) LCN-2 fecal levels after infection. *, *P* < 0.05 (dZD versus dZD plus ETEC and dPD versus dPD plus ETEC). (B) MPO fecal levels after infection. *, *P* < 0.05 (HC versus HC plus ETEC and dPD versus dPD plus ETEC); **, *P* < 0.01 (dZD versus dZD plus ETEC) (*n* = 8/group).

We have previously demonstrated the tight correlation of fecal biomarkers lipocalin-2 (LCN-2) and myeloperoxidase (MPO) with malnutrition and disease burden in the MAL-ED study in children (19, 20). As shown in Fig. 2, fecal levels were significantly elevated in mice at day 3 following a single dose of ETEC for both LCN-2 (*, *P* < 0.05 [dZD versus dZD plus ETEC and dPD versus dPD plus ETEC]) (Fig. 2A) and MPO (*, *P* < 0.05; **, *P* < 0.01 [dZD versus dZD plus ETEC]) (Fig. 2B) (*n* = 8/group). The exceptionally high levels of LCN-2 and MPO in the zinc-deficient infected mice led us to investigate potential zinc effects on ETEC virulence traits.

### Influence of zinc on ETEC H10407 growth and virulence gene expression *in vitro*.

**FIG 3:**
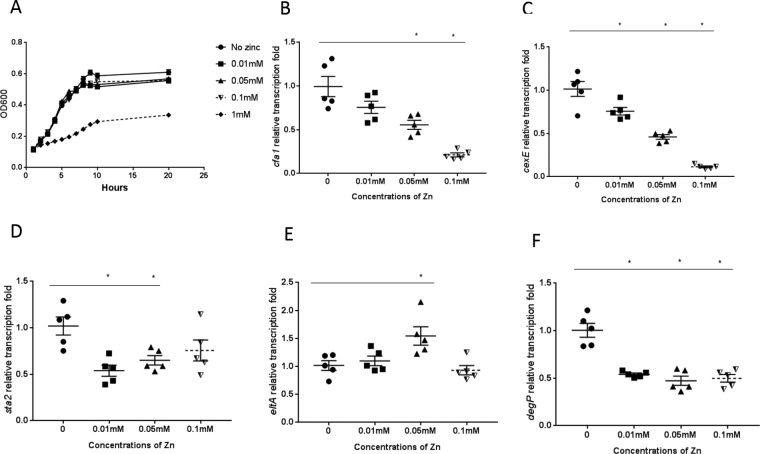
Influence of zinc on ETEC growth and virulence gene expression *in vitro*. (A) Growth of ETEC strain H10407 in the presence of zinc. Bacteria were incubated with select concentrations of zinc and monitored spectrophotometrically. *, no zinc versus 1 mM zinc; OD600, optical density at 600 nm. (B to F) Bacterial mRNA was isolated from ETEC strain H10407 after incubation with non-growth-inhibitory zinc concentrations. Reverse transcription-qPCR (RT-qPCR) was performed for measurement of expression of specific genes (*cfaA*, *cexE*, *sta2*, *eltA*, and *degP*). *, *P* < 0.05 (comparing the findings seen with zinc exposure to those seen in the absence of zinc exposure) (*n* = 5/group).

We have previously seen effects of zinc on enteroaggregative E. coli (EAEC) virulence trait expression (21, 22). To investigate zinc effects on ETEC virulence, we conducted similar *in vitro* growth assays. As shown in Fig. 3, supplementation of zinc at a concentration of 0.01 mM or higher (the concentrations were not inhibitory for ETEC growth until >0.1 mM was reached) (Fig. 3A) was able to alter expression of the *cfaA*, *cexE*, *sta2*, *eltA*, and *degP* genes (*, *P* < 0.05 for comparisons of the findings obtained with bacteria that had been exposed to zinc with those obtained with bacteria that had not been cultured with zinc) (*n* = 5/group) (Fig. 3B to F).

### Zinc supplementation enables catchup growth in ETEC H10407-infected groups, without decreasing intestinal colonization.

**FIG 4:**
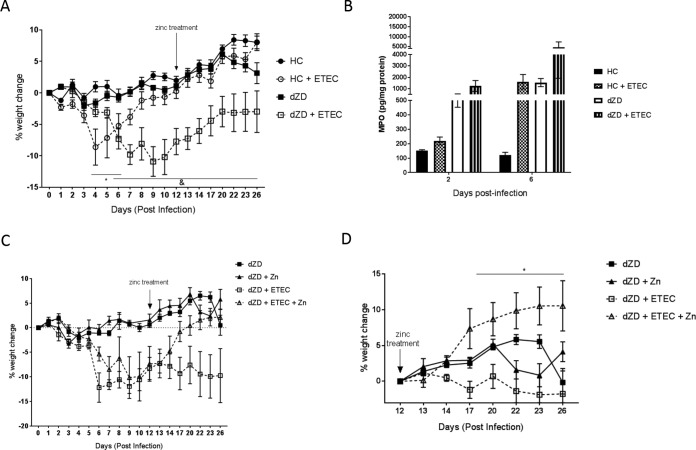
Zinc supplementation enables catchup growth in zinc-deficient ETEC H10407-infected C57BL/6 mice. Zinc-deficient mice received supplemental zinc sulfate in drinking water (150 mg/liter) starting from day 12 post-ETEC infection. Daily weight measures were collected from the day of infection until the end of the experiment. (A) Percentages of body weight change using day 0 as the baseline without discriminating the zinc treatment groups. *, *P* < 0.05 (for HC versus HC plus ETEC during days 4 to 6 postinfection and for dZD versus dZD plus ETEC during days 6 to 26 postinfection). (B) Myeloperoxidase (MPO) fecal levels after infection. Protein extracts were obtained from fecal samples at day 2 postinfection by lysing fecal specimens in RIPA buffer. Supernatants from the lysates were used for protein quantification and specific ELISAs for evaluation of MPO fecal levels. (C) Percentages of body weight change using day 0 as the baseline and discrimination of the zinc-treated groups. (D) Percentages of body weight change determined using day 12 (the initial day of zinc treatment) as the baseline. *, *P* < 0.0001 (dZD plus ETEC versus dZD plus WT plus Zn during days 23 to 26 postinfection) (*n* = 4/group).

At day 12 postinfection, zinc in the drinking water (150 mg/liter) was given to zinc-deficient infected mice. Figure 4A shows percentages of body weight changes during the whole experiment (before and after zinc treatment start) without discriminating the results obtained with infected zinc-deficient mice from those obtained with the mice that had received zinc. Increased fecal MPO levels were observed at day 6 pi in both “house chow” (a defined protein source diet without zinc)-deficient and zinc-deficient infected mice (Fig. 4B). By day 17 (5 days after zinc treatment), catchup growth had been enabled in the infected zinc-supplemented mice (*P* < 0.05) (Fig. 4C). Further, the significance of the benefit of zinc supplementation reached a *P* value of <0.0001 for percent body weight change values compared with those determined for the non-zinc-supplemented groups starting at day 12 as the baseline (when zinc supplementation was implemented; *n* = 4/group) (Fig. 4D).

**FIG 5:**
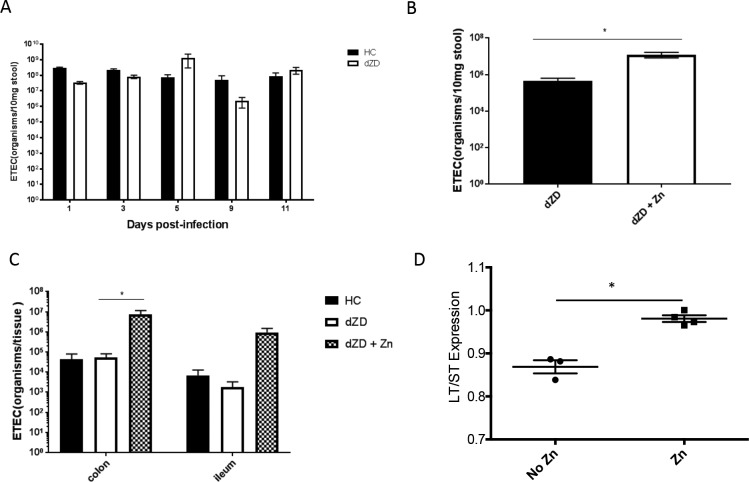
Zinc supplementation increases ETEC H10407 intestinal colonization in zinc-deficient infected C57BL/6 mice. Zinc-deficient mice received supplemental zinc sulfate in drinking water (150 mg/liter) starting from day 12 post-ETEC infection. Quantitative PCR was performed using DNA samples that had been extracted from stool samples or intestine samples from ETEC-infected mice. (A) Stool ETEC H10407 shedding before zinc treatment. (B) Effect of zinc treatment on stool ETEC H10407 shedding after 13 days of zinc treatment (day 25 postinfection). *, *P* < 0.05 (dZD versus dZD plus Zn). (C) Effect of zinc treatment on colon and ileum ETEC H10407 colonization after 13 days of zinc treatment (day 25 postinfection). *, *P* < 0.05 (dZD versus dZD plus Zn in the colon) (*n* = 4/group). (D) Effect of zinc treatment on cecal ETEC H10407 toxin expression following zinc treatment. *, *P* = 0.0008 (no Zn versus Zn).

Quantitative PCR was performed on DNA that was extracted from stool samples or intestine samples from ETEC-infected mice. Interestingly, zinc supplementation significantly increased both ETEC shedding and the intestinal burden in stool samples (Fig. 5). ETEC H10407 possesses both the *sta2* and *eltA* genes, encoding ST and LT, respectively. Considering that zinc downregulated *sta2* but upregulated *eltA in vitro*, this suggests that ST may be more highly associated with inflammation and weight loss and that LT is more highly associated with colonization. Hence, the specific roles of ST and of LT require further investigations. As shown in Fig. 5D, the ratio of LT expression to ST expression was altered in the cecum of zinc-deficient mice receiving zinc treatment.

### The effects of zinc deficiency and zinc supplementation are toxin specific.

**FIG 6:**
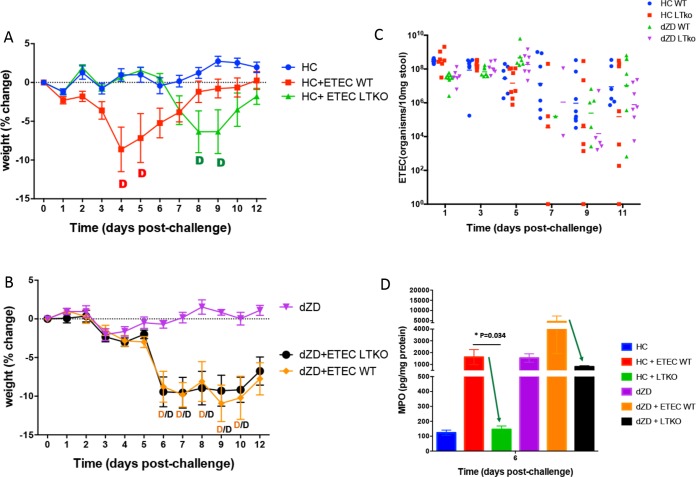
Outcomes of infection with an ETEC LT mutant of strain H01407 (LTKO) are different from those seen with the wild type in house chow-deficient and zinc-deficient mice. House chow-deficient or zinc-deficient mice were infected with either the ETEC H10407 wild type or ETEC LTKO strains. Daily weight measures were collected from the day of infection until the end of the experiment. (A) Percentages of body weight change of house chow mice after infection. (B) Percentages of body weight change of zinc-deficient mice after infection. *, *P* < 0.05 (ZD versus ZD plus ETEC WT/LTKO). (C) Shedding of ETEC WT and LTKO strains in HC- and dZD-fed mice. LTKO ETEC showed a trend of lower shedding levels than WT ETEC following 1 week of infection in both diet groups. There were no significant differences between groups. (D) Myeloperoxidase (MPO) fecal levels after 6 days of infection. Protein extracts were obtained from fecal samples at day 2 postinfection by lysing fecal specimens in RIPA buffer. Supernatants from the lysates were used for protein quantification and specific ELISAs for evaluation of MPO. *, *P* < 0.05 (HC plus ETEC WT versus HC ETEC LTKO) (*n* = 8/group).

In order to dissect the different roles of ST and LT in our infection model, we created an LT-negative (LT^−^) (i.e., LT knockout [LTKNO]) mutant of H10407. LTKO-infected mice fed a standard diet had a delayed disease outcome similar to what was observed in zinc-deficient mice infected with wild-type (WT) H10407 (Fig. 6A), while zinc-deficient mice infected with LTKO had a disease outcome similar to that seen with wild-type-infected zinc-deficient mice (Fig. 6B). The levels of shedding of the organisms were not significantly different (Fig. 6C). LTKO-infected zinc-deficient mice showed lower levels of fecal MPO than ETEC wild-type control-challenged mice (Fig. 6D).

**FIG 7:**
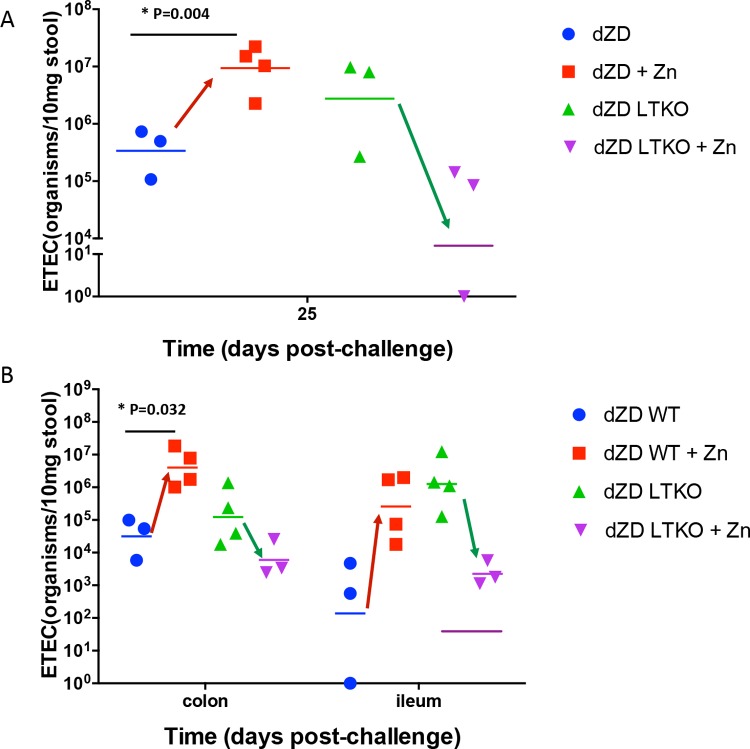
Zinc supplementation decreases intestinal colonization by the ETEC LT mutant of H010407 (LTKO) in zinc-deficient infected C57/BL6 mice. Zinc-deficient mice received supplemental zinc sulfate in drinking water (150 mg/liter) starting from day 12 post-ETEC infection. Quantitative PCR was performed using DNA samples that had been extracted from stool samples or intestine samples from infected mice. (A) Effect of zinc supplementation on stool ETEC LTKO shedding after 13 days of zinc treatment (day 25 postinfection). (B) Effect of zinc supplementation on colon and ileum ETEC LTKO colonization after 13 days of zinc treatment (day 25 postinfection) (*n* = 4/group).

Zinc supplementations of zinc-deficient mice infected with either WT or LTKO ETEC showed very different responses. Whereas zinc supplementation increased shedding and the ileal burden of WT ETEC, zinc reduced shedding and the tissue burden of LTKO ETEC (Fig. 7). These data suggest that, while zinc supplementation may significantly reduce the expression and impact of ST as demonstrated *in vitro* and in the LTKO-infected mice, zinc supplementation has mixed effects on LT- and ST-expressing WT ETEC. Further studies on additional wild-type, knockout, and single-toxin-expressing strains are needed to evaluate the specific impact of zinc deficiency as well as of zinc supplementation on ETEC toxins.

In conclusion, we describe a robust C57BL/6 mouse model of ETEC infection with measurable outcomes of diarrhea, weight loss, stool shedding, tissue burden, and fecal biomarkers. This model will be useful in vaccine development as well as for further investigation into the impact of dietary deficiencies such as zinc deficiencies or of novel interventions on ETEC disease outcomes.

## DISCUSSION

The need for robust and affordable animal models for studying enteric pathogens is urgent for developing and evaluating novel interventions, including therapeutics or vaccines. ETEC plays a major role in the burden of moderate to severe diarrhea as recently reported in a large multicenter study in developing countries (GEMS) (3). The potential scope of its role in milder diarrhea in community-based studies has been extended in the multicountry MAL-ED study as well (2).

Considering the frequency of causation of mild to severe diarrhea by ETEC worldwide and, from the EAEC experience, its potential to obtain additional virulence traits (23, 24), it is of critical importance that it remain a focus of vaccine development. This illustrates the need for a consistently reproducible small-animal model to test therapeutic interventions and vaccines. In this report, we demonstrate a C57BL/6 mouse model of enteropathy, with or without acute, watery diarrhea, with oral infections with ETEC strain H10407, which expresses both ST and LT as well as the CFA/1 colonization factor.

Other groups have previously used murine models as an approach for evaluating ETEC pathogenesis (25, 26). However, none have evaluated the effects of undernutrition on infection susceptibility and outcomes. Here, we examined weight, biomarker, and diarrhea outcomes with ETEC infection in the context of protein and zinc deficiencies, two common deficiencies in children in developing countries (27, 28). Differential nutritional status can modulate intestinal microbiome, metabolome, and immune responses (29–31), although these effects are currently being defined only in the context of enteric infections. Furthermore, undernourished children in low-resource settings are associated with poor vaccine responses but the mechanisms of the responses are not fully understood (32).

Antibiotics are widely used to disrupt intestinal microbiota and induce susceptibility to infection and to develop animal models for several enteropathogens in murine and other systems (33–35). Allen and colleagues reported the use of streptomycin in drinking water for a mouse model of ETEC infection, in addition to the use of cimetidine to reduce gastric acidity. Interestingly, they observed only intestinal colonization (24 h after infection) with no signs of disease or of an inflammatory response (25). In our study, we used an antibiotic cocktail which has been used for other enteropathogen mouse models by us and other groups (21, 36). This approach allowed us to see significant disease outcomes after ETEC infection and robust stool shedding for at least 5 days or longer postinfection. Different antibiotic treatments likely lead to different susceptibility conditions for the host (37). ETEC infection without antibiotic pretreatment did not induce infection or disease even in undernourished mice.

In recent years, we have gained better appreciation of the potential impacts and physiologic effects of enteric infections beyond just transient overt diarrhea (2, 20, 38). To these clinical studies, we can now add an experimental model of ETEC infections that includes growth impairment and intestinal inflammation in addition to overt watery diarrhea. Regarding growth, it is now recognized that infections by several enteropathogens, such as enteroaggregative E. coli, Campylobacter jejuni, and Giardia sp., can be associated with deficits in growth and, potentially, in cognitive development, even without causing diarrhea (39–42). Intestinal inflammation likely plays an important role in these effects (43). Although a neonatal mouse model showed death outcomes within 24 h postinfection (44), our weaned-mouse model and dietary differences can model acutely symptomatic as well as “silent” (with respect to overt diarrhea) ETEC infections given orally. Other published ETEC models that showed disease consequences (i.e., not just intestinal colonization) did not use oral administration of the bacterial inoculum (26, 45), limiting their direct clinical relevance. The model we describe here closely reflects the route of infection and potentially relevant outcomes of ETEC infection seen in humans. The growth impairment and diarrhea induced by oral administration of ETEC strain H10407 represent robust outcomes that match those specific consequences from clinical studies of ETEC infections (3, 46, 47).

In an effort to establish an affordable and reproducible ETEC infection model, we thought it important to demonstrate colonization and diarrhea in mice fed a standard rodent chow diet (HC). Unlike the dPD and dZD diets, the standard rodent chow is a very common diet produced by Harlan Teklad that is used by Jackson Laboratories. We have also used a defined protein source control diet (Research Diets), which produced results similar to those produced by the HC model (data not shown). As demonstrated previously (21), feeding with a zinc-free protein source (e.g., egg white protein) diet for 2 weeks is required in order to achieve “clinical” zinc deficiency. While this is not nearly as affordable or rapid a model as the HC model, it has been very useful in determining the effects of zinc deficiency on host microbiome and metabolome (29) as well as the effects of zinc deficiency and subsequent zinc supplementation on virulence traits of diarrheagenic bacteria (21, 22). Interestingly, we have seen only modest alterations in resident microbiotas of mice in the 2-week period of dZD feeding required to generate zinc deficiency (29). However, note that we have not extensively investigated the effects of our antibiotic cocktail (required for colonization in this model) on resident microbiota or metabolome or whether these effects are altered by protein or zinc deficiencies. Studies to address these important issues are under way.

In the current study, infected protein-deficient mice were the only infected mice that exhibited growth deficits in the absence of overt diarrhea. In addition, the greatest growth impairments induced by ETEC were seen with nourished but zinc-deficient mice. Increased fecal MPO and LCN-2 levels were seen for all infected groups regardless of the diets used. However, the strongest induction was seen with zinc-deficient mice. Both host and pathogen mechanisms affected by zinc deficiency might contribute to this outcome. Interestingly, when zinc was provided to mice in the zinc-deficient infected groups, the mice underwent catchup growth. This finding corroborates clinical studies that noted the growth-promoting effects of zinc (48) and further helps to model the benefits underlying the use of zinc for treatment of infectious diarrhea in zinc-deficient subjects.

Previous studies by our group and others have shown the antivirulence effects of zinc on several bacteria, including diarrheagenic E. coli (22, 49, 50). In the context of ETEC, zinc was already shown to inhibit ion secretion induced by LT through targeting the cyclic AMP-stimulated potassium channel (51). In addition, zinc was shown to inhibit ETEC K88 adhesion *in vitro* (52). However, the influence of zinc on ETEC virulence gene expression is still unclear. In order to test this, we assessed virulence gene expression of the ETEC H10407 strain incubated with a range of subinhibitory and inhibitory concentrations of zinc *in vitro*. Downregulation of the CFA/I colonization factor was observed.

Expression levels of the *cfaI* and *cexE* genes were decreased by administration of zinc in a dose-dependent manner. Interestingly, both genes are regulated by *cfaD*, a homolog of *aggR* in EAEC, a major transcriptional regulator. These findings suggest that zinc acts on ETEC virulence gene expression in a manner similar to that in which it influences EAEC virulence gene expression, as previously described (53). However, while zinc decreased ST gene expression, it did not decrease LT gene expression. This result shows that ST expression is more altered by zinc than LT expression (which tended to actually be increased with low zinc levels) and might help to explain our *in vivo* observations. Nourished mice infected by LTKO had delayed disease compared to the wild-type infection, suggesting an impaired ability of ETEC to colonize the intestinal epithelium in early infection (∼1 to ∼5 days pi), although the level of impairment was not maintained throughout the experiment. Other *in vivo* models have shown the importance of LT for colonization (25, 45). LT was also important for inducing fecal MPO, and the data further corroborate the importance of LT for ETEC pathogenesis. Interestingly, the influence of LT on the ETEC zinc deficiency model was not evident regarding clinical outcomes and is likely due to the significant impact of zinc deficiency on ST and potentially on other virulence traits in the pathogen and possibly in part on host immune responses (21, 54). However, zinc supplementation decreased colonization in mice infected with LTKO but not in the ETEC-wild-type-infected mice, which also suggested that zinc can even increase expression of LT. Specifically, ETEC strains producing ST are associated with moderate to severe diarrhea and increased risk of death in children (2), and this effect may account for the decreased diarrhea outcomes associated with zinc supplementation (55). On the other hand, LT^−^ ETEC may be associated with environmental enteropathy, as suggested by *in vitro* studies (56). Our results suggest that the consequences of infections by ETEC strains, including both ST^−^ ETEC and LT^−^ ETEC strains, especially in malnourished children, warrant further assessment.

In conclusion, our findings demonstrate the establishment of a new mouse model of ETEC infection that shows growth impairment, diarrhea, and intestinal inflammation across different host nutritional states, which are features commonly seen in children in developing areas. Zinc is clearly an important determinant of clinical outcomes and differentially influences ETEC gene virulence expression. The clinically relevant outcomes seen in this murine model, which mimic several outcomes in children with ETEC infections, help advance our understanding of ETEC pathobiology and provide key tools for testing of new interventions, such as potential vaccine candidates.

## MATERIALS AND METHODS

### Animal husbandry.

This study included the use of mice. This study was carried out in strict accordance with the recommendations included in the Guide for the Care and Use of Laboratory Animals of the National Institutes of Health. The protocol was approved by the Committee on the Ethics of Animal Experiments of the University of Virginia (protocol number 3315). All efforts were made to minimize suffering. This protocol was approved by the Institutional Animal Care and Use Committee of the University of Virginia and is in accordance with their policies. The University of Virginia is accredited by the Association for the Assessment and Accreditation of Laboratory Animal Care, International (AAALAC). Mice used in this study were male 22-day-old C57BL/6 strain mice from Jackson Laboratories (Bar Harbor, ME). The mice weighed approximately 11 g on arrival and were cohoused in groups with up to five animals per cage. The vivarium was kept at a temperature of between 68 and 74°F, with 14-h light and 10-h dark cycles.

### Rodent diet.

Weaned mice (22 days old) were acclimated, fed a regular diet for 2 to 5 days, and then fed a Harlan Laboratories (Indianapolis, Indiana) standard rodent “house chow” (HC) diet, a defined protein source diet without zinc (dZD), or a defined reduced-protein diet (2%) (dPD) from Research Diets (New Brunswick, New Jersey). All diets were isocaloric, and calories from fat, protein, and carbohydrates were as previously reported (29). We have previously demonstrated serum and tissue zinc deficiency in mice fed the dZD diet for 14 days (21). For experiments in which zinc supplementation was employed, zinc sulfate was dissolved in water and filtered before being provided in drinking water at 150 mg/liter. This concentration was based on the estimated dose/weight approximation equivalence of the U.S. recommended daily allowance for zinc for children (57).

### Antibiotic disruption of native flora.

Four-week-old C57Bl/6J mice were placed on a standard rodent chow (HC) diet, a zinc-deficient diet (dZD), or a protein-deficient diet (dPD) for 2 weeks, following a 2-day acclimation period upon arrival. On day 10 of diet feeding, mice were given gentamicin (35 mg/liter), vancomycin (45 mg/liter), metronidazole (215 mg/liter), and colistin (850 U/ml) in drinking water to disrupt resident microbiota as previously published (21, 33). After 3 days on antibiotics in water, mice were given untreated water for 1 day and then were given a single oral challenge by gavage of 10^9^ CFU ETEC–100 μl Dulbecco's modified Eagle's medium (DMEM) or DMEM without bacteria as a control (described below).

### ETEC infection.

Enterotoxigenic Escherichia coli (H10407, a prototype strain that produces both ST and LT) was originally isolated from a patient with severe, cholera-like watery diarrhea in Bangladesh (7, 53). Cultures were grown from glycerol stocks in DMEM at 37°C in a shaking incubator. Each infected mouse received an inoculum of ∼1 × 10^9^ CFU ETEC in 100 μl of freshly prepared DMEM; controls received 100 μl of DMEM alone.

In this study, we used the following six groups for each experiment: mice fed HC with or without ETEC, mice fed dPD with or without ETEC, and mice fed dZD with or without ETEC. Each experiment used 8 mice per experimental group, and mice were euthanized at either day 3 or day 10 postinfection.

### Protein extraction from stool and tissue.

After rapid dissection of the mouse intestines, cecal contents and stool samples were flash frozen in liquid nitrogen (LN2). At the time of assay, samples were lysed in radioimmunoprecipitation assay (RIPA) buffer (20 mM Tris [pH 7.5], 150 mM NaCl, 1% Nonidet P-40, 0.5% sodium deoxycholate, 1 mM EDTA, 0.1% SDS) containing protease inhibitor cocktail (Roche) and phosphatase inhibitors (1 mM sodium orthovanadate, 5 mM sodium fluoride, 1 mM microcystin-LR, and 5 mM beta-glycerophosphate). Tissue lysates were cleared by centrifugation, and the supernatant was used for total protein measurements, cytokine measurements by Luminex assay (Bio-Rad), and specific enzyme-linked immunosorbent assays (ELISAs) for evaluation of lipocalin-2 (LCN-2) and myeloperoxidase (MPO) levels as previously described (29).

### DNA extraction.

DNA was isolated from fecal pellets using a QIAamp DNA stool minikit as previously described (58). DNA was extracted from frozen tissue samples using a QIAamp DNA tissue kit (Qiagen). To enhance extraction of the pathogen's DNA, we made an improvement in the original protocol, namely, vigorous homogenization of the samples with 300 mg of 1.0-mm-diameter zirconia beads (BioSpec, Bartlesville, OK, USA) using a Mini-BeadBeater (BioSpec, Bartlesville, OK, USA). DNA from cecum or stool content was extracted from the thawed stool samples using a QIAamp DNA stool kit (Qiagen), following the manufacturer's instructions. After extraction, DNA was eluted in 200 μl elution buffer and stored at −20°C.

### Real-time PCR for ETEC quantification.

Stool DNA and tissue were analyzed for the ETEC-specific LTb and STh genes to determine the levels of shedding of the organism in stool and the burden in the tissue as described by Liu et al. (15).

Quantification of the infection was performed in a Bio-Rad CFX PCR detection system by interpolating threshold cycle (*C_T_*) values of each run with a standard curve of known amounts of ETEC DNA, and the data were transformed into numbers of organisms per milligram of sample. The master mix solution and primers were used as described elsewhere (3). Amplification consisted of 3 min at 95°C, followed by 40 cycles of 10 s at 95°C and 30 s at 58°C.

### *In vitro* gene expression analysis.

Total cellular RNA was obtained from ETEC cultures using RNeasy kits (Qiagen), and cDNA was synthesized from 1 μg RNA using iScript (Bio-Rad). For quantitative PCR analyses of virulence factor mRNA abundance, cDNA was diluted 1:8; 4 μl of this dilution was used for each PCR. Bioline Sensi-Fast Sybr reagent was used for quantitative PCRs. The primer sequences used are listed in Table S1 in the supplemental material. The PCR conditions were as follows: 95°C for 5 min followed by 40 cycles of 95°C for 15 s and 60°C for 30 s followed by a melt curve analysis. Data were analyzed and are presented based on the relative-expression method (59). The formula used for the calculation was as follows: relative expression = 2^−(SΔ*CT* − CΔ*CT*)^, where Δ*CT* is the difference in threshold cycle values between the gene of interest (i.e., the LTb gene) and the control gene (i.e., the 16S gene). In this formula, “S” represents E. coli-challenged mice and “C” represents uninfected mice.

### Statistical analysis.

Data analyses were performed with GraphPad Prism 6 software (GraphPad Software). All statistical analyses were done from raw data with the use of analysis of variance, Student *t* tests, and Bonferroni *post hoc* analysis where applicable. Differences were considered significant at a *P* value of <0.05. Data are represented as means ± standard errors of the means.

## Supplementary Material

Supplemental material
