# Supplementary material for: Critical Role of Zinc in a New Murine Model of Enterotoxigenic Escherichia coli Diarrhea
Source: Infect Immun. 2018 Jun 21;86(7):e00183-18. doi: 10.1128/IAI.00183-18 (PMC6013668; doi:10.1128/IAI.00183-18)
Supplement: Supplemental material [file supp_86_7_e00183-18__index.html]

Supplemental material 

# Critical Role of Zinc in a New Murine Model of Enterotoxigenic Escherichia coli Diarrhea

## Supplemental material

- Supplemental file 1 -

  Table S1. Primer sequences.

  PDF, 28K
