# Supplementary material for: Critical Role of Zinc in a New Murine Model of Enterotoxigenic Escherichia coli Diarrhea
Source: Infect Immun. 2018 Jun 21;86(7):e00183-18. doi: 10.1128/IAI.00183-18 (PMC6013668; doi:10.1128/IAI.00183-18)
Supplement: Supplemental material [file IAI.00183-18_zii999092441s1.pdf]

Supplemental Table 1

Primer sequences

Gene target	Sequences
sta2	F: 5'-3' CACCCGGTACAAGCAGGATT
	R: 5'-3' TTCACCTTTCGCTCAGGATG
eltA	F: 5'-3' CAAAGCCGGTTTGTGTTCCT
	R: 5'-3' TGCTGACTCTAGACCCCCAGA
cfa1	F: 5'-3' TCAGTGTGTCATGGGGAGGA
	R: 5'-3' ACCGGCAGTTTTAGGTGCAG
cexE	F: 5'-3' AGTCCGATGCATGGCTACAC
	R: 5'-3' TGGCTATGGGGTCTCTCTCC
degP	F: 5'-3' CTGACCCTGGGCTTACTGCG
	R: 5'-3' CCACGCCCTG ATCTTTGCCT
